# Plausible Sources of Membrane-Forming Fatty Acids on the Early Earth: A Review of the Literature and an Estimation of Amounts

**DOI:** 10.1021/acsearthspacechem.2c00168

**Published:** 2022-12-22

**Authors:** Zachary R. Cohen, Zoe R. Todd, Nicholas Wogan, Roy A. Black, Sarah L. Keller, David C. Catling

**Affiliations:** ^†^Department of Chemistry, ^‡^Department of Earth and Space Sciences, and ^§^Astrobiology Program, University of Washington, Seattle, Washington 98195, United States

**Keywords:** Fatty acid, membrane, prebiotic chemistry, origin of life, astrobiology

## Abstract

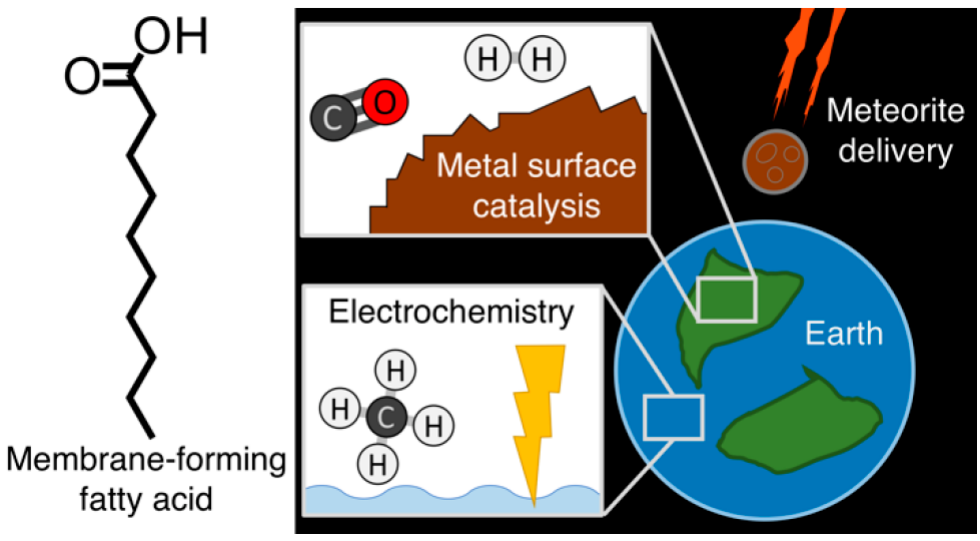

The first cells were plausibly bounded by membranes assembled from fatty acids with at least 8 carbons. Although the presence of fatty acids on the early Earth is widely assumed within the astrobiology community, there is no consensus regarding their origin and abundance. In this Review, we highlight three possible sources of fatty acids: (1) delivery by carbonaceous meteorites, (2) synthesis on metals delivered by impactors, and (3) electrochemical synthesis by spark discharges. We also discuss fatty acid synthesis by UV or particle irradiation, gas-phase ion–molecule reactions, and aqueous redox reactions. We compare estimates for the total mass of fatty acids supplied to Earth by each source during the Hadean eon after an extremely massive asteroid impact that would have reset Earth’s fatty acid inventory. We find that synthesis on iron-rich surfaces derived from the massive impactor in contact with an impact-generated reducing atmosphere could have contributed ∼10^2^ times more total mass of fatty acids than subsequent delivery by either carbonaceous meteorites or electrochemical synthesis. Additionally, we estimate that a single carbonaceous meteorite would not deliver a high enough concentration of fatty acids (∼15 mM for decanoic acid) into an existing body of water on the Earth’s surface to spontaneously form membranes unless the fatty acids were further concentrated by another mechanism, such as subsequent evaporation of the water. Our estimates rely heavily on various assumptions, leading to significant uncertainties; nevertheless, these estimates provide rough order-of-magnitude comparisons of various sources of fatty acids on the early Earth. We also suggest specific experiments to improve future estimates. Our calculations support the view that fatty acids would have been available on the early Earth. Further investigation is needed to assess the mechanisms by which fatty acids could have been concentrated sufficiently to assemble into membranes during the origin of life.

## Introduction

Cells use bilayer membranes to separate themselves from their environment. In modern cells, these membranes are composed of phospholipids. During the origin of cells on Earth, more primitive membranes likely played a similar role,^1^ sequestering cellular building blocks^2,3^ and polymers.^4^ The hydrocarbons in modern phospholipids are tails of fatty acids connected by an ester linkage to the glycerol backbone. Fatty acids themselves can assemble into membranes. Fatty acids were likely more abundant than phospholipids on the early Earth, leading to a common hypothesis that the membranes of the first cells were composed of fatty acids.^1^

**Figure 1:**
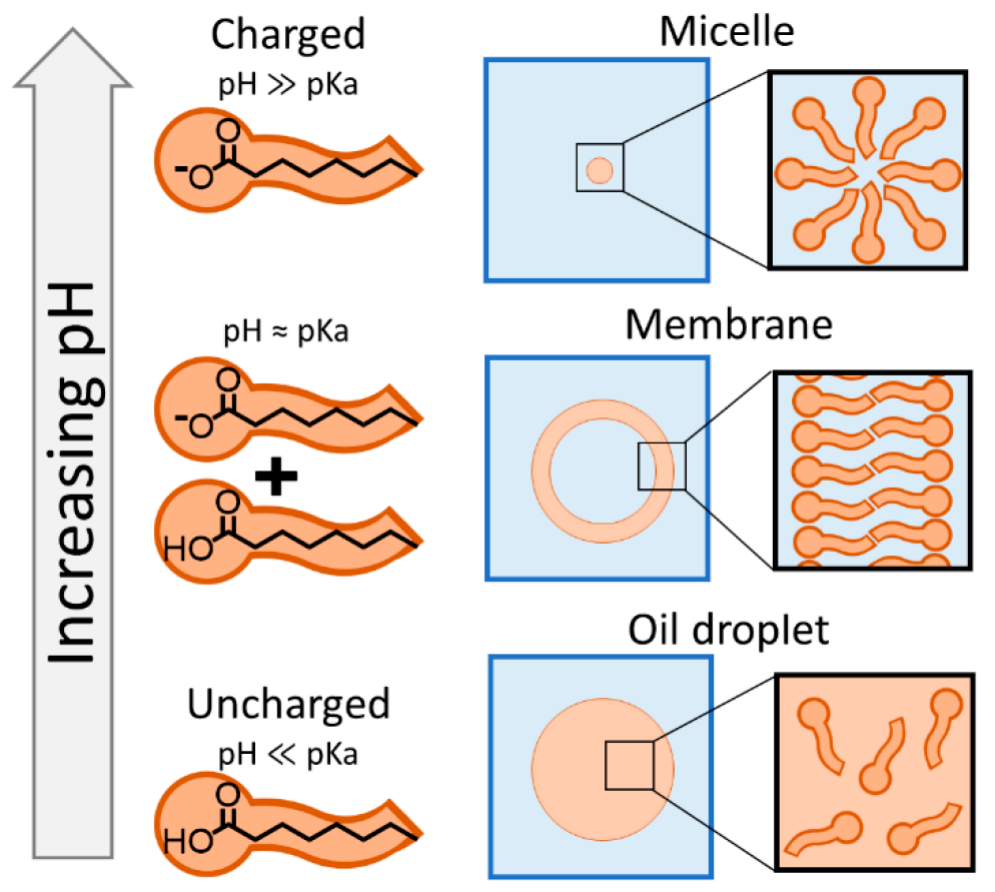
Fatty acid assembly depends on the pH of the surrounding solution. When the pH is below the effective p*K*_a_ of the fatty acids in a bilayer, fatty acids form an oil that is immiscible with the surrounding aqueous solution (bottom). When the pH is near the effective p*K*_a_, fatty acids assemble into bilayers in a membrane (middle). Vesicles, spherical shells of these membranes, may have served as the membrane compartments for the first cells on Earth. When the pH is above the p*K*_a_ of the fatty acids in a bilayer, the fatty acids assemble into micelles, which cannot encapsulate aqueous solutes (top).

Fatty acids consist of a hydrocarbon tail and a carboxylic acid headgroup (Figure 1). Saturated fatty acids with eight or more carbons in a linear chain can assemble into membranes.^5^ Fatty acids with unsaturated^6^ or branched^7^ chains can also assemble into membranes, although in these cases it is unknown whether the minimum number of carbons for membrane assembly is greater or less than eight. Fatty acids with carboxylic acids at both ends of a carbon chain do not assemble into membranes on their own, although these dicarboxylic acids can incorporate into membranes when additional types of amphiphiles are present.^8^

To form membranes, the solution pH must be within about half a unit of the effective p*K*_a_ of the fatty acid in a bilayer^5^ (where p*K*_a_ = −log_10_ of the equilibrium constant, *K*_a_, for the dissociation of the fatty acid into a proton and the negatively charged amphiphile). If the solution pH is high enough such that the vast majority of headgroups are charged or if the fatty acids have fewer than eight carbons, then fatty acids assemble into nanoscale micelles that cannot encapsulate aqueous solutes (Figure 1). On the other hand, if the solution pH is low enough that the vast majority of headgroups are uncharged, then fatty acids separate into an oil phase.^9^

Fatty acid membranes provide some advantages for early cell replication compared to modern phospholipid membranes.^9^ For example, fatty acid membranes are moderately permeable to salts and small organic molecules such as nucleotides, allowing internal replication of nucleic acids, which would have been critical for developing cells.^10^ The surface area of these vesicles increases when they incorporate additional fatty acids from the environment into the membrane.^11,12^ A growing vesicle can be supplied with fatty acids from micelles^11,13^ or from other vesicles.^14^ Vesicles that retain fatty acids grow while others shrink, which could have enabled competition between primitive cells for a limited supply of fatty acids.^12^ After acquiring excess membrane surface area, primitive cells could have divided when exposed to modest shear stress.^13,15^ Vesicles of phospholipids do not grow or divide as readily because aqueous solubility of a phospholipid with two hydrophobic tails is much lower than the solubility of a single-tailed fatty acid, so transfer through aqueous solution is slower.^16,17^

**Figure 2:**
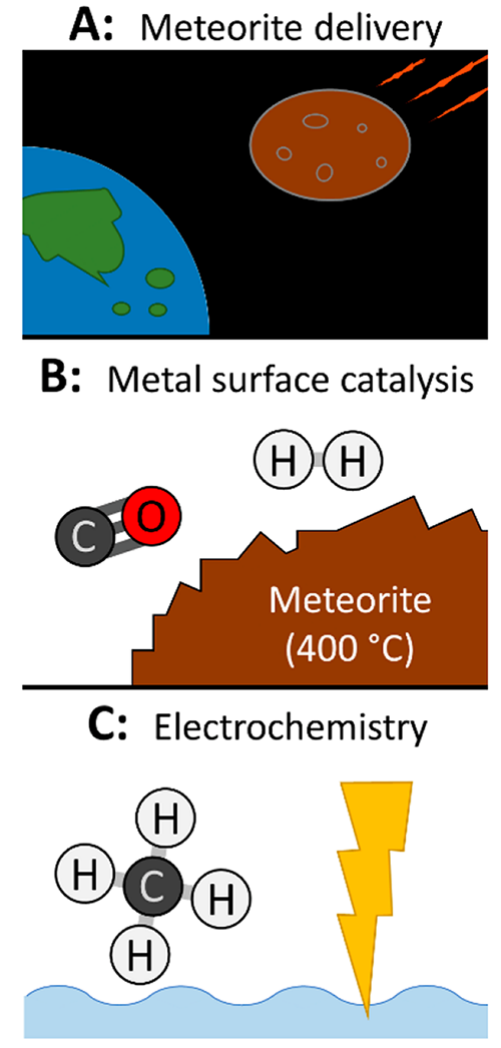
There are three well-characterized sources that could have provided fatty acids to the early Earth. (A) Carbonaceous meteorites can deliver fatty acids.^19−22^ (B) Metal surfaces can catalyze fatty acid synthesis. As one example, Nooner and Oro mixed filings of the Canyon Diablo meteorite (containing iron and nickel) with deuterium and carbon monoxide gases, and the mixture was heated to 400 °C to produce fatty acids.^23^ Similar experiments have used pure Fe, Ni, or Fe- and Ni-containing minerals as catalysts and a variety of carbon and hydrogen sources to synthesize fatty acids (Table 1). (C) Fatty acids can also be synthesized during electrical sparking (Table 2). As one example, Yuen et al. used an electric discharge to synthesize fatty acids from methane.^24^

Here, we review how membrane-forming fatty acids could have been supplied on the early Earth. We identify three relatively well-characterized sources of abiotic fatty acids: delivery by meteorites, synthesis on the surface of metal catalysts, and synthesis by electrochemistry (Figure 2). There are also reports of fatty acid syntheses that fall outside these categories, but these have been less robustly investigated. We summarize the important details of the experiments that have been carried out and discuss whether similar reactions could have plausibly occurred on the early Earth. Finally, we estimate the total mass of fatty acids produced from each of the three relatively well-characterized sources during the Hadean eon when the first cells are hypothesized to have formed.^18^

## Carbonaceous Meteorites Deliver Fatty Acids to Earth

Fatty acids have been detected in a variety of carbonaceous meteorites that have landed on Earth,^19−22^ and molecules extracted from at least one such meteorite assemble into membranes.^25^ At least 18 different carbonaceous meteorites have been analyzed, and both linear-chain and branched-chain fatty acids have been identified.^26^ Depending on the type of carbonaceous meteorite, the abundance of membrane-forming fatty acids can range from 1 ppb to 100 ppm by mass.^26^ Recent reviews provide detailed analyses of meteoritic fatty acids.^26,27^ Importantly, it remains unclear which types of reactions are responsible for synthesizing meteoritic fatty acids in space.^26^

Could fatty acids delivered by carbonaceous meteorites have dissolved into water and assembled into membranes? Meteorites can fragment in airbursts during passage through the atmosphere, allowing some fatty acids to remain intact.^28,29^ Meteorites with radii less than 100 m tend to fragment when differential pressure across the small body exceeds the material strength.^28^ The Chelyabinsk ordinary chondrite meteorite (radius ∼ 10 m) that fell in Russia in 2013 fragmented at an altitude above 25 km, and fragments were spread into an area 250–300 km^2^ around the trajectory.^30^ The largest fragment was ∼0.7 m mean diameter and fell into a lake. Fatty acids in carbonaceous chondrites would likely be similarly dispersed over a wide spatial area.

When meteorite fragments disperse into water on Earth’s surface, fatty acids can dissolve. Numerical models suggest that nucleobases leach out of 20 cm meteorite fragments and mix into the surrounding water within about three years.^29^ At moderately alkaline pH, fatty acids can be even more soluble because of their charged headgroup. However, in order for fatty acids to assemble into membranes, the fatty acids must accumulate in solution above the critical vesicle concentration (∼15 mM for decanoic acid^3^). If fatty acids are present at concentrations below the critical vesicle concentration, membrane assembly does not occur.

Here, we use the measured abundance of decanoic acid (a 10-carbon fatty acid) in carbonaceous meteorites^26^ to calculate the volume of a meteorite fragment that would be required to deliver enough decanoic acid into water so that the concentration of decanoic acid equals the critical vesicle concentration. Although it is not known precisely how meteorite size, initial velocity, or impact angle influences the fraction of fatty acids that survive impact, we note that a large portion of the meteorite’s initial mass (and thus a large portion of its fatty acids) may be destroyed by ablation and heating during travel through the atmosphere.^31−33^ Given that our estimates rely on the average mass fraction of decanoic acid that has been recovered from natural carbonaceous meteorites and that we do not know a priori the mass, velocity, and impact angle of each meteorite, we assume the measured fatty acid abundances represent the average survival over the entire population of possible impacts. Although this assumption introduces potential errors, a more precise calculation is beyond the scope of this study.

The volume of a meteorite fragment (*V*_meteor_, expressed in m^3^) that would be required to deliver enough decanoic acid to reach the critical vesicle concentration as a function of water volume (*V*_water_, expressed in m^3^) is given by eq 1:
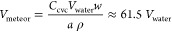
1where *C*_cvc_ is the critical vesicle concentration for decanoic acid (15 mol/m^3^ of water, equivalent to 15 mM), *w* is the molar mass of decanoic acid (0.1723 kg/mol), *a* is the dimensionless fraction of the meteorite’s mass that is decanoic acid (2 × 10^–5^ for CM2 type meteorites), and ρ is the meteorite density (2100 kg/m^3^ for CM type meteorites).

**Figure 3:**
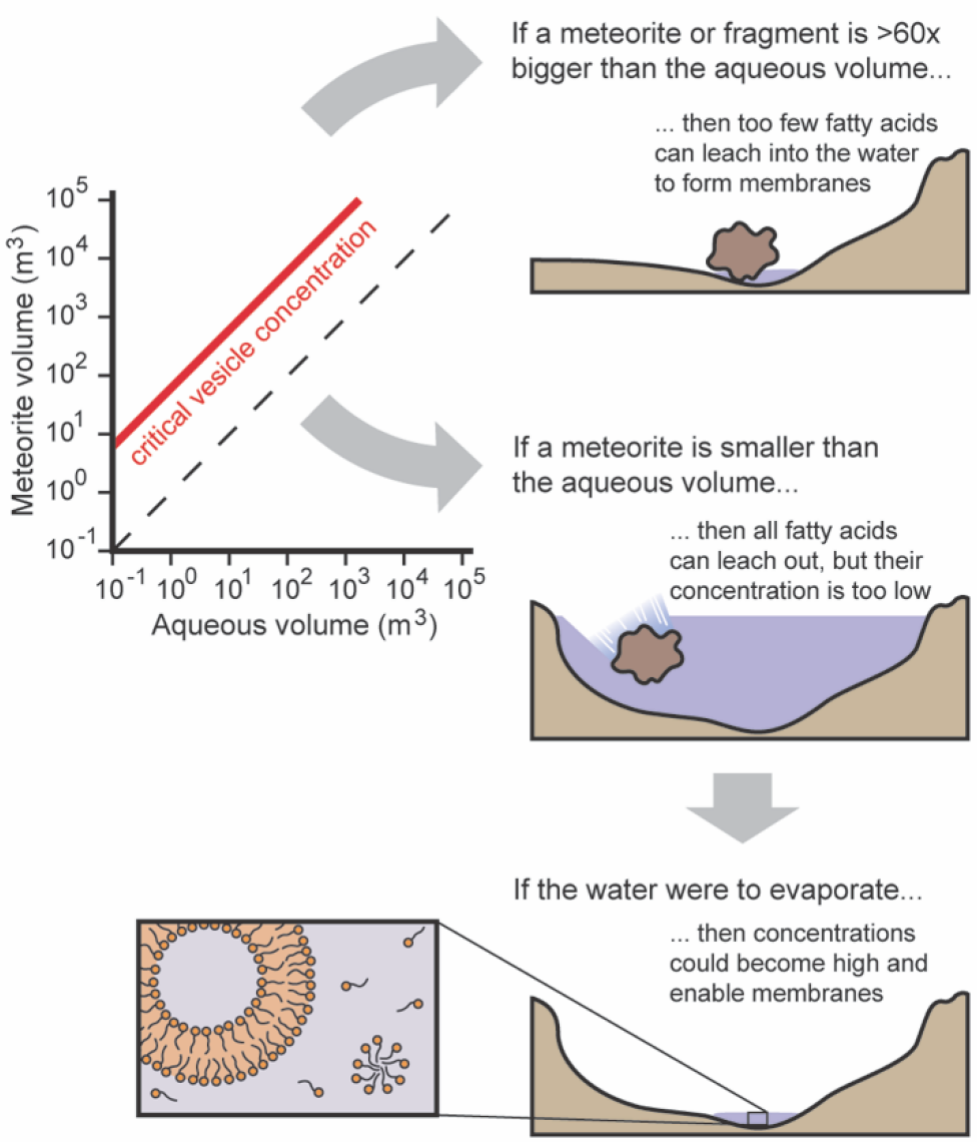
A single fragment of a carbonaceous meteorite cannot directly deliver enough decanoic acid to a body of water to form membranes. To exceed the critical vesicle concentration (∼15 mM),^3^ the volume of the meteorite (red line) would exceed the volume of the water. However, subsequent evaporation of the water could concentrate decanoic acid and enable membrane formation. A CM2 type meteorite is assumed because it contains the most decanoic acid on average (20 ppm by mass^26^). The density of CM-type meteorites is 2100 kg/m^3^.^38^ Only meteorites with radii less than 100 m (∼10^6^ m^3^ for a spherical meteorite) can fragment and impact the Earth’s surface with low enough energy to preserve fatty acids.^28,29^ See eq 1 for details of the calculation.

The result of our estimate is shown in Figure 3. To deliver enough decanoic acid to form membranes, the volume of the meteorite fragment would have to exceed the volume of the waterbody. If a meteorite fragment were to land in a large enough waterbody to submerge the fragment, the decanoic acid that subsequently dissolved in the water would be too dilute to form membranes. However, this does not rule out membrane formation. The concentration of fatty acids could increase during dry periods, accompanied by a net loss of water due to evaporation.^34^ Although we limited our calculation to decanoic acid because the critical vesicle concentration has been measured, a range of fatty acids from 8 to 12 carbons can be delivered simultaneously during a meteorite impact. The presence of these additional fatty acids could enable membrane formation at lower concentrations of decanoic acid.^35,36^ We do not consider that CM type meteorites can contain significant water (up to ∼9% by mass^37^). If all meteoritic fatty acids were to somehow dissolve in only this meteoritic water, the concentration of decanoic acid could exceed the critical vesicle concentration; this can be interpreted as an upper limit for the concentration of fatty acids delivered by a meteorite.

To conclude our section on carbonaceous meteorites, we find that meteorite delivery was unlikely to directly yield high enough aqueous concentrations of fatty acids to form membranes on the early Earth. Additional processes would have been necessary to further concentrate fatty acids above the critical vesicle concentration, which we cannot rule out.

## Catalysis of Fatty Acid Synthesis by Metal Surfaces

**Figure 4:**
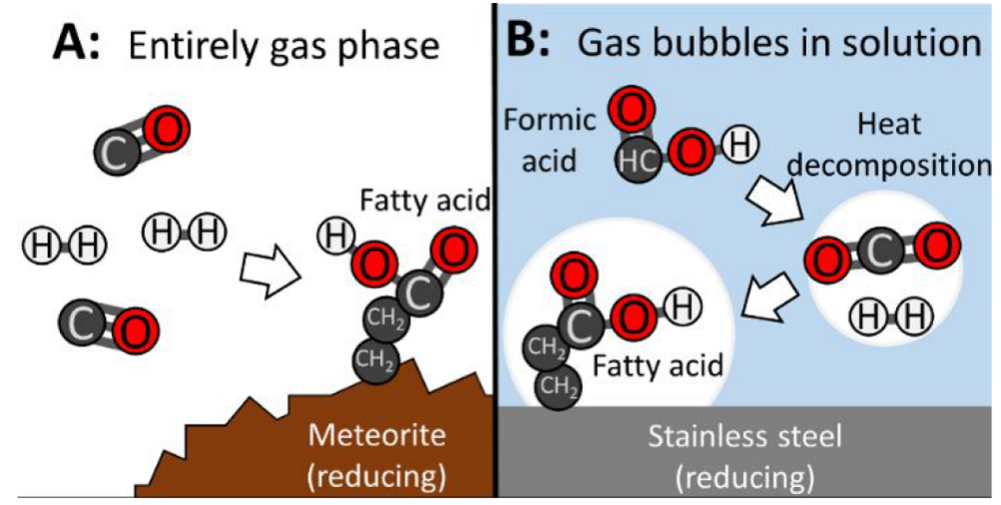
Fatty acid synthesis occurs at the interfaces between reducing metal surfaces and a gaseous headspace. (A) Nooner and Oro showed that deuterium and carbon monoxide gases react together on the surface of hot (400 °C) meteorite filings to produce membrane-forming fatty acids.^23,50^ When the meteorite filings were artificially oxidized, fatty acid synthesis was not observed.^23^ (B) In hydrothermal experiments, McCollum et al. report that the synthesis of membrane-forming fatty acids occurs within gaseous bubbles adsorbed onto oxidation-resistant stainless steel surfaces.^49^ When oxidized metal surfaces are present instead of stainless steel, only short-chain (<5 carbons) fatty acids are formed.^23,45−48^ In these hydrothermal experiments, aqueous formic acid or oxalic acid is used for experimental convenience as a source of H_2_ and CO_2_.

The most commonly reported abiotic synthesis of fatty acids involves catalytic metal surfaces. Within this class of syntheses, Fischer–Tropsch reactions are the most thoroughly investigated. Fischer–Tropsch reactions occur when H_2_ and either CO or CO_2_ adsorb onto a metal surface.^39,40^ Surfaces of solid iron or nickel are most commonly tested, although FeS, NiS, and Fe_3_O_4_ minerals have also been used to produce fatty acids.^41−44^ In most experiments, metal surfaces must be heated above 150 °C to produce fatty acids. Catalysts contain metals in their reduced form; the synthesis of membrane-forming fatty acids (at least 8 carbons) has not been demonstrated on oxidized metal surfaces.^23,45−48^ The synthesis of fatty acids seems to occur at the gas–solid interface (Figure 4). Even in experiments designed to eliminate gaseous headspace, reactions are suggested to proceed in gaseous bubbles within the aqueous solution.^48^ Whether or not an explicit gaseous headspace is present, the carbon-containing precursors are generally supplied as gases. However, for experimental convenience in hydrothermal experiments, both formic acid and oxalic acid have been used as aqueous starting materials for fatty acid synthesis because both compounds decompose into H_2_ and CO_2_ at temperatures above 150 °C.^49^

**Figure 5:**
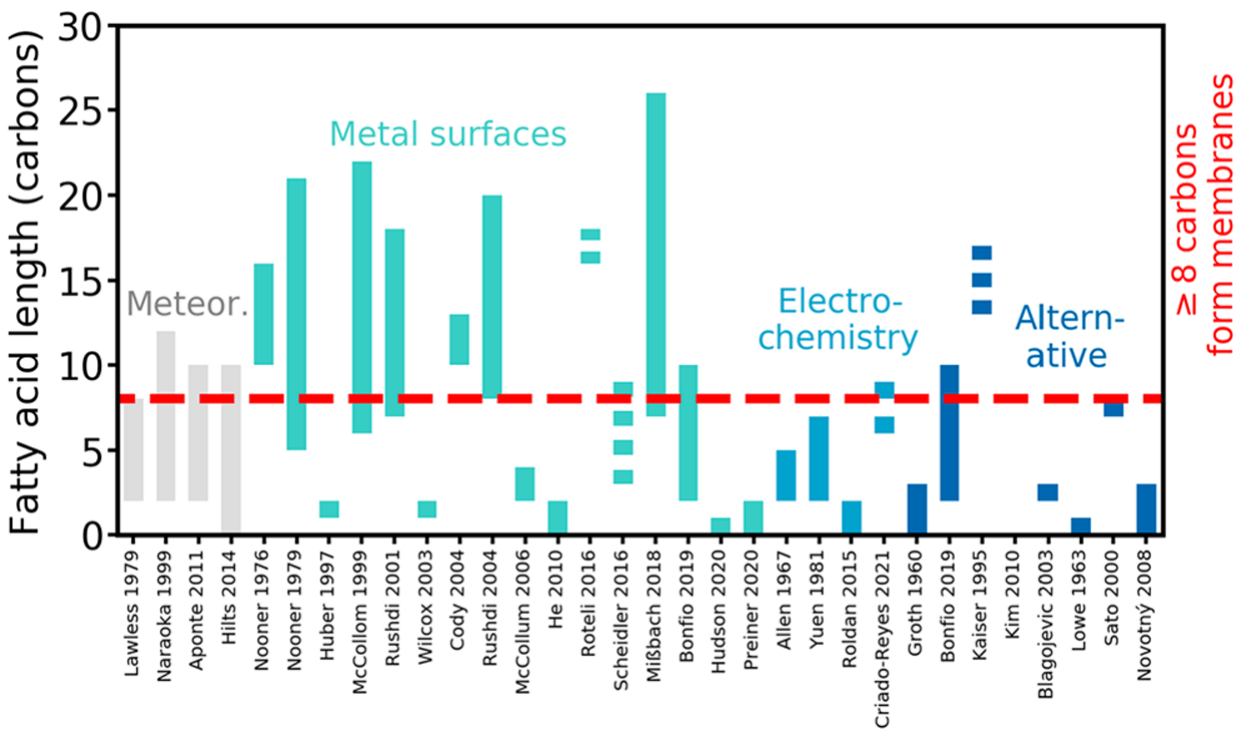
Length of fatty acids delivered by meteorites (labeled “Meteor.”) or produced in abiotic synthesis experiments. Fatty acids with at least eight carbons, indicated by the dashed red line, can assemble into membranes. All the fatty acids produced are saturated and unbranched (except in Scheidler et al. 2016, where experiments also produced unsaturated fatty acids). Note that detection of a fatty acid with a certain length may not have been attempted during every experiment.

Metal-catalyzed reactions generally create a diverse mixture of product types, including hydrocarbons and fatty alcohols in addition to fatty acids.^48,49,51−54^ For each type of product, molecules with more carbons are less abundant.^40^ Many experiments have produced only short-chain carboxylic acids containing fewer than the 8 carbons required for membrane assembly^5^ (Figure 5). The carbon chain of a fatty acid could be elongated upon further reaction with a metal catalyst,^40^ although additional experiments are required to validate this hypothesis. Table 1 summarizes experiments in the literature that have produced fatty acids using metal catalysts.

**Table 1:** Summary of Experiments That Used Metal Surfaces to Catalyze the Synthesis of Fatty Acids^a^

year and ref	explicit gas phase?	explicit aqueous phase?	H_2_ source	carbon source	solid surface	reaction conditions	number of carbons per fatty acid
1976^50^	Yes	No	D_2_ (g)	CO (g)	Meteorite filings and K_2_CO_3_	50 h at 370 °C	10–16
1979^23^	Yes	No	D_2_ (g)	CO (g)	Meteorite filings (containing iron and nickel) and carbonate salts	48 h at 400 °C	5–21
1997^41^	Yes	Yes	N/A	CO (g) and CH_3_SH (aq)	NiS–FeS	1 week at 100 °C	2
1999^49^	Yes	Yes	Formic acid or oxalic acid (aq)	Formic acid or oxalic acid (aq)	Stainless steel	>48 h at 175 °C	6–22
2001^51^	No	Yes	Oxalic acid (aq)	Oxalic acid (aq)	Stainless steel	48 h at 100 °C	7–18
2003^63^	Yes	No	N/A	CH_4_ and CO_2_	5% Pt/alumina	2 h with temperature increasing from 200 to 400 °C	2
2004^64^	No	Yes	Formic acid (aq)	Nonane-thiol	Ni^0^^b^	6 h at 250 °C and 200 MPa	10–13
2004^52^	No	Yes	Oxalic acid (aq)	Oxalic acid (aq)	Stainless steel	18 h at 300 °C	8–20
2006^48^	No	Yes	Formic acid (aq)	Formic acid (aq)	Powdered iron	86 h at 250 °C and 325 bar	2–4
2010^65^	Yes	Yes	Water (l)^c^	CO_2_ (aq)	Iron nanoparticles	25–200 h at fixed temp from 80 to 200 °C	1–2
2016^66^	Yes	Yes	N/A	Formamide	Meteorite powder	24 h at 140 °C	16, 18
2016^42^	Yes	Yes	N/A	CO (g) and acetylene (g)	NiS	1 week at 105 °C and 2.5 bar	Saturated: 3, 5
							Unsaturated: 3, 5, 7, 9
2018^53^	No	Yes	Oxalic acid (g)	Oxalic acid (g)	Stainless steel	>66 h at 175 °C	7–26
2018^67^	Yes	Yes	N/A	CO_2_ (g)	Fe^0^, Ni^0^, Co^0^	16 h at fixed temp from 30 to 150 °C	1–2
2019^54^	Yes	Yes	N/A	HCN, Na_2_CO_3_, formaldehyde	Macroporous Ni	Multistep reaction	2–10
2020^43^	No	Yes	Water (l)	CO_2_ (aqueous)	NiS–FeS	50 min at room temperature	1
2020^44^	Yes	Yes	H_2_ (g)	CO_2_ (g)	Fe_3_S_4_, Fe_3_O_4_, Ni_3_Fe	0–24 h at fixed temp from 60 to 100 °C	1–2

a Unless otherwise specified, fatty acids were saturated and unbranched. Note: The minimum and maximum length fatty acids may have been synthesized during separate experiments that were reported in the same reference.

b Other minerals were tested as well, although reactions with Ni^0^ produced linear fatty acids with the greatest number of carbons.

c H_2_ is generated when water oxidizes the Fe.

Metal-catalyzed reactions could have occurred on the early Earth after meteorite impacts, which delivered iron, nickel, and heat. Meteoritic metals could have been exposed to atmospheric H_2_, CO_2_, and CO, enabling surface-catalyzed synthesis of fatty acids. Extremely large impactors, about the size of the asteroid Vesta (∼10^20^ kg), could have transformed the Earth into a global Fischer–Tropsch reactor with surface temperatures >100 °C and high partial pressures of H_2_, CO_2_, and CO for thousands of years.^55^ Catalytic metal surfaces would be required to produce fatty acids, so future research in this area will be especially valuable if it constrains the location and quantity of reduced metals after such impacts.^56^ Although a large impact would have been catastrophic for any life that was already present on Earth, it could have potentially seeded Earth’s postimpact surface with fatty acids and other necessary biomolecules, enabling life to subsequently emerge.^57^

Ground-breaking experiments by Nooner and Oro modeled a postimpact scenario for fatty acid synthesis.^23,50^ It remains uncertain how the yield of fatty acids depends on experimental parameters such as partial pressure and temperature. Kinetic models for the Fischer–Tropsch process have been developed in industrial settings, which generally do not mimic plausible early Earth conditions, and the quantitative form of the models depends on the design of the reactor.^58^ Until experiments are conducted to understand how fatty acid production depends on reaction parameters more relevant to the early Earth, there will be substantial uncertainty in estimates of production of fatty acids by metal catalysts on the early Earth.

Another natural setting in which the metal catalyzed synthesis of fatty acids might occur is in a hydrothermal environment, where the conversion of CO_2_ and H_2_ into fatty acids is thermodynamically favorable.^59^ Fatty acids have been detected in natural hydrothermal systems; however, it is unclear what fraction of those fatty acids were produced by modern cells.^60^ Ultramafic rocks (relatively Fe- and Mg-rich and Si-poor) are common at some deep sea hydrothermal vents.^61^ Could metal-rich minerals within these rocks serve as catalysts for fatty acid synthesis? To address this question, we consider the oxidation state of the mineral surface. Ultramafic mineral surfaces become oxidized by reacting with seawater and generating H_2_ in serpentinization reactions.^62^ As noted above, to date, there have been no reports of the synthesis of membrane-forming fatty acids (at least 8 carbons) on oxidized metal surfaces.^23,45−48^ Laboratory experiments failed to produce fatty acids with more than 2 carbons when oxidized olivine (ultramafic mineral with general composition (Fe,Mg)SiO_4_) was the sole catalyst.^45^ In these experiments, the olivine was heated in water for 96 days to allow ample time for oxidation by serpentinization, and formic acid was included as an additional source of H_2_.^45^ It is unknown whether olivine surfaces could catalyze fatty acid synthesis before becoming oxidized during serpentinization.

Hydrothermal experiments with oxidation-resistant stainless steel surfaces, clearly not natural settings, do produce fatty acids from formic acid with up to 22 carbons.^49^ However, these experiments also permitted vapor-phase reactions, which may have enabled production of longer fatty acids regardless of the oxidation state of the surface. Thus, in natural hydrothermal settings, it remains unclear if catalytically active, reduced mineral surfaces could persist or be replenished quickly enough to catalyze fatty acid synthesis. Without a suitable catalyst, fatty acid synthesis would likely be slow or nonexistent in hydrothermal environments.^62^ In conclusion, additional experiments are necessary to determine whether fatty acids could be synthesized in natural hydrothermal settings.

## Electrochemical Synthesis of Fatty Acids

Electrochemical reactions can generate diverse organic compounds, including amino acids,^68,69^ nucleobases,^70^ and fatty acids. For example, spark discharges between an electrode in the gaseous headspace and another electrode either in the same headspace^71^ or in solution^24,72^ can produce fatty acids. In the experiments summarized in Table 2, CH_4_ in the gaseous headspace served as the source of carbon in three of the four experiments generating linear fatty acids of varying lengths with only one experiment reporting chains long enough (at least 8 carbons) to form membranes.^71^ An additional electric discharge experiment produced carbon chains of 2–6 carbons with carboxylic acids on both ends.^73^

**Table 2:** Summary of Electrochemical Experiments That Have Synthesized Fatty Acids^a^

year and ref	electric discharge description	carbon source	reaction conditions	number of carbons per fatty acid
1967^72^	1 electrode in solution, 1 electrode in headspace	CH_4_ (g)	96 h of discharge	2–5
1981^24^	1 electrode in solution, 1 electrode in headspace	CH_4_ (g)	24 h of discharge; pH = 8	2–7
2015^78^	Both electrodes in solution	CO_2_ (aq)	150 h with electric potential cycling from −0.8 to 0.2 V	1–2
2021^71^	Both electrodes in headspace	CH_4_ (g)	Discharge alternating on/off for 14 days, pH = 8.7, room temperature	6, 9

a All fatty acids were saturated and unbranched.

The goal of most electrochemical experiments is to simulate lightning strikes through a methane-rich atmosphere on the early Earth. However, attributes of natural lightning strikes are challenging to reproduce in the laboratory. Criado-Reyes et al. used a Tesla coil that generated a 3 × 10^4^ V potential for 7 days at room temperature.^71^ In contrast, a natural lightning strike generates a potential of about 10^8^ V for <1 s,^74^ and temperatures of the air surrounding a lightning strike can reach 30 000 °C.^75^ Unfortunately, experimental evidence is lacking to describe how fatty acid yields depend on the voltage, duration, or total energy dissipated by laboratory sparking. Chyba and Sagan assumed that electrochemical production of organic molecules on early Earth should depend on the total amount of electrical energy dissipated by lightning strikes and coronal discharges.^76^ Additional experiments are needed to validate this assumption.

Only slightly more is known about the role of the atmosphere and solid surfaces during electrochemical synthesis. Experiments by Schlesinger and Miller have shown that the yield of amino acids during sparking increases with the partial pressure of CH_4_;^77^ similar experiments with fatty acids are still needed. Borosilicate glass as a substrate has also been shown to increase the yields of electrochemical fatty acid synthesis.^71^ Although borosilicate does not occur naturally, silicates would have been ubiquitous on the early Earth because they are common rock-forming minerals. Additional experiments are necessary to better understand how the yields of electrochemical fatty acid production depend on the gaseous, solid, aqueous, and electrical environments. Nevertheless, the available experimental data suggest that fatty acid synthesis is possible under certain electrochemical conditions.

## Alternative Types of Fatty Acid Synthesis

**Figure 6:**
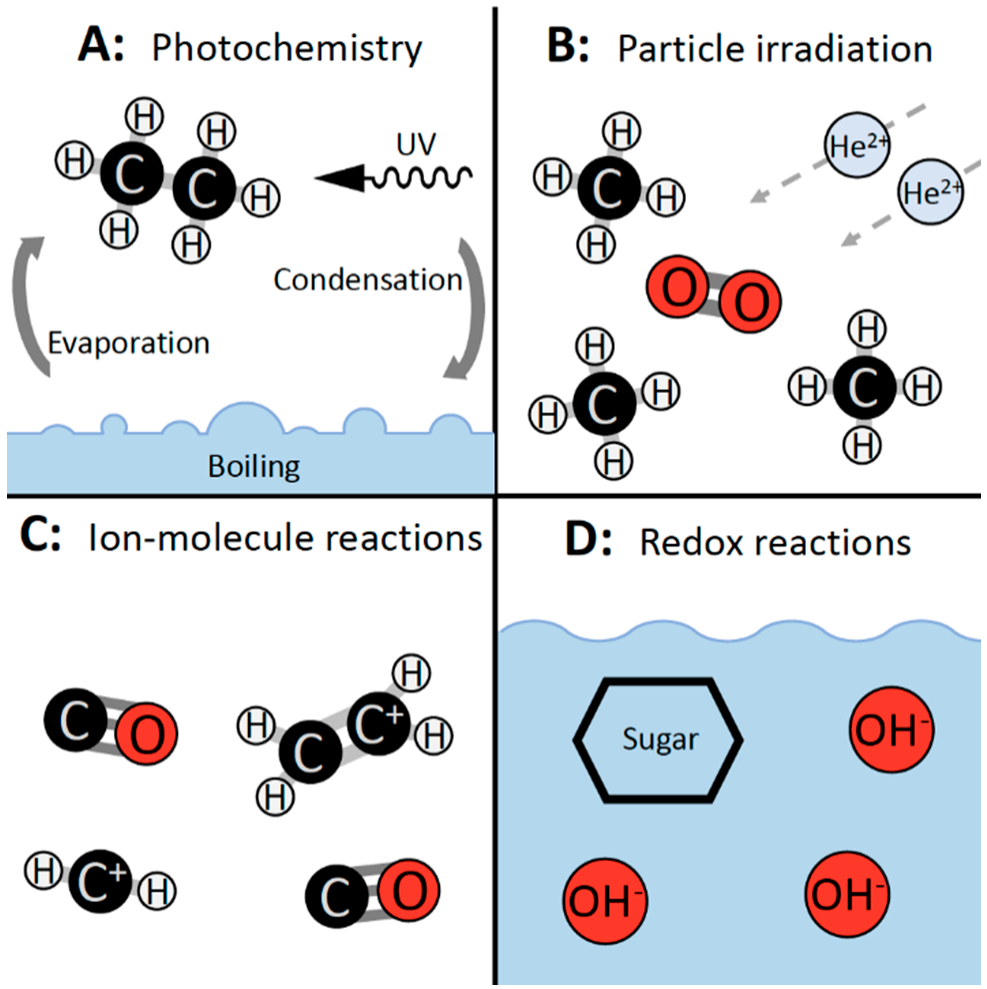
Alternative reaction types that have demonstrated fatty acid synthesis. (A) Groth and Weyssenhoff used UV photochemistry to convert ethane into fatty acids.^79^ (B) Kaiser et al. irradiated an ultracold (10 K) mixture of ∼99% CH_4_ and ∼1% O_2_ with 9 MeV alpha particles to produce fatty acids.^80^ (C) Blagojevic et al. report reactions between gas-phase ions (CH_2_^+^ and C_2_H_4_^+^) and CO to produce fatty acids.^82^ (D) Novotný et al. report the decomposition of monosaccharides into fatty acids under mild alkaline conditions (50 mM NaOH).^84^

In addition to the three main sources reviewed above, there are references in the literature to four alternative types of fatty acid synthesis (Figure 6, Table 3). These include photochemical reactions,^54,79^ irradiation by massive particles,^80,81^ gas-phase ion–molecule reactions,^82^ and redox reactions in aqueous solution.^83−85^

**Table 3:** Summary of Experiments That Have Synthesized Fatty Acids by the Mechanisms Illustrated in Figure 6^a^

year and ref	reactants	reaction conditions	number of carbons per fatty acid
**Photochemistry**			
1960^79^	Ethane, NH_3_, H_2_O	1 week of UV irradiation (185 and 254 nm)	1–3
2019^54^	HCN, formaldehyde, Na_2_CO_3_	Multistep reaction (254 nm UV irradiation and catalysis by Ni surface)	2–10
			
**Irradiation by Massive Particles**			
1995^80^	CH_4_, O_2_	Irradiation with 9 MeV alpha particles at 10 K	13, 15, 17
2010^81^	CO_2_, hydrocarbons (1–6 carbons)	Irradiation with 5 keV electrons at 10 K	undetermined
			
**Gas-Phase Ion–Molecule Reactions**			
2003^82^	CO and either CH_2_^+^ or C_2_H_4_^+^	CH_4_ and C_2_H_4_ are ionized and reacted together in the presence of He and trace H_2_O at 0.35 Torr and room temperature^b^	2–3
			
**Redox Reactions in Solution**			
1963^83^	HCN, NH_3_	90 °C for 18 h	1
2000^85^	Fatty aldehydes (either 7 or 8 carbons)	30% H_2_O_2_ solution at 90 °C for 2 h	7 or 8
2008^84^	Glucose, fructose, arabinose, glyceraldehyde, or dihydroxyacetone	50 mM NaOH for 1 h	1–3

a All fatty acids were saturated and unbranched.

b The authors suggest that their synthesis models low temperature and low pressure environments.

There have been two reports of fatty acid synthesis by UV photochemistry. By irradiating a gaseous mixture of ethane and ammonia above boiling water (Figure 6A) at 185 and 254 nm, Groth and Weyssenhoff generated fatty acids 1–5 carbons long.^79^ Bonfio et al. used a multistep procedure to synthesize longer-chain fatty acids capable of membrane assembly.^54^ UV irradiation at 254 nm was used in one step, and catalysis by a nickel surface was used in a subsequent step. Photochemistry could provide a plausible explanation for the presence of fatty acids on the surface of the early Earth because UV radiation down to ∼200 nm could have penetrated prebiotic atmospheres.^86^ The experiments by Groth and Weyssenhoff did not generate fatty acids when methane was used instead of ethane;^79^ further investigation is needed to determine whether radiation from realistic early Earth solar spectra could have enabled photochemical conversion of methane into fatty acids. In contrast, Bonfio et al. used starting materials that are more plausibly prebiotic such as formaldehyde, HCN, NaH_2_PO_4_, Na_2_CO_3_, and NaSH,^54^ but these reagents impose a constraint on the geochemical scenario for the early Earth. In a separate experiment by Dworkin et al., membrane-forming amphiphiles were synthesized by irradiating ultracold ices composed of 100:50:1:1 H_2_O:CH_3_OH:NH_3_:CO with 121.6 and ∼160 nm UV photons, although the identity of these amphiphiles was not determined.^87^

The second type of “alternative” fatty acid synthesis is irradiation by particles. Experiments of this type were designed to simulate chemistry in the outer solar system, rather than the early Earth. By irradiating an ultracold (10 K) mixture of ∼99% CH_4_ and ∼1% O_2_ with 9 MeV alpha particles, Kaiser et al. produced linear fatty acids with 13, 15, or 17 carbons^80^ (Figure 6B). In a subsequent experiment by Kim and Kaiser, an ultracold (10 K) mixture of CO_2_ and hydrocarbons (1–6 carbons) was irradiated with 5 keV electrons. The presence of carboxylic acids was confirmed by Fourier-transform infrared spectroscopy, but specific fatty acids were not identified.^81^

A third alternative fatty acid synthesis is discussed by Blagojevic et al.^82^ In their experiments, reactions between gas-phase ions (CH_2_^+^ and C_2_H_4_^+^) and molecules (CO) resulted in carboxylic acids with 2 or 3 carbons (Figure 6C). Additional gas-phase ion–molecule reactions to produce formic acid (e.g., CH_4_ + O_2_^+^ → HCOOH_2_^+^ + H) have been suggested but not validated experimentally.^88^ These reactions were suggested to occur in the interstellar medium, and the relevance to early Earth conditions has not been explored.

The fourth group of lesser studied fatty acid syntheses are redox reactions. Three sets of redox reactions have been shown to produce fatty acids in solution. The first experiments from Sato et al. use hydrogen peroxide to oxidize fatty aldehydes (7–8 carbons) to produce fatty acids with the same carbon-chain length.^85^ It is unlikely that sufficient hydrogen peroxide would have been present on the early Earth.^89^ In the second set of experiments, Novotný et al. report the decomposition of monosaccharides (3–6 carbons) into linear carboxylic acids (1–3 carbons) under mild alkaline conditions (50 mM NaOH, Figure 6D).^84^ Diverse monosaccharides are obtained in low yield during the formose reaction,^90^ and alkaline lakes on early Earth might have been sites for decomposition into short-chain fatty acids.^91,92^ Finally, Lowe et al. reported production of formic acid and potentially other carboxylic acids by heating a mixture of ammonia and hydrogen cyanide to 90 °C.^83^ Hydrogen cyanide is considered a prebiotic reagent that can be sequestered and concentrated as ferrocyanide within early Earth environments.^91,93^

There are numerous additional pathways for the synthesis of short-chain (1–3 carbons) linear fatty acids,^94^ but because membrane assembly requires fatty acids with at least 8 carbons, we have generally omitted them. We are unaware of any abiotic mechanisms by which carbon chains of existing fatty acids are elongated. Modern cells synthesize fatty acids from acetyl-CoA using the sophisticated enzyme complex fatty acid synthetase.^95^ Because the intermediate compounds that are produced during this process are unstable, it is believed that fatty acid synthesis via these reactions would have occurred at a negligible rate on the early Earth before the emergence of enzymes.^96^ Finally, thermodynamic calculations suggest that fatty acids can be synthesized from polyaromatic hydrocarbons,^97^ but to our knowledge, this synthesis has never been demonstrated experimentally.

## Critical Analysis of Analytical Techniques

Many of the reactions discussed above produce a wide variety of fatty acids and other products. Uniquely identifying products can be challenging, and determining the concentration of each product is even more difficult. In Table 4, we summarize the reported concentrations of fatty acids from each experiment and comment on potential limitations of the analyses. In general, the papers in Table 4 convincingly identify fatty acids of various lengths but do not provide substantial evidence for the fatty acid concentrations that they report. Moreover, many papers report only relative concentrations of fatty acids (e.g., *X*% of all products by mass or moles) instead of absolute concentrations (e.g., *X* moles or *X* grams), so it is difficult to compare product yields between experiment types. An ideal strategy for the characterization and absolute quantitation of fatty acids was employed by Yuen et al., which involved chromatography and tandem mass spectrometry with isotope-labeled internal standards to eliminate matrix effects.^24^ Additional synthesis experiments that determine the absolute concentration of fatty acids would be valuable.

**Table 4:** Summary of Analytical Techniques in Each Report^a^

year and ref	carbons per fatty acid	yield information	analytical techniques	identification of products	quantitation of product concentrations
**Method 1: Metal Surface Catalysis**					
1976^50^	10–16	None	GC-MS	Unambiguous identification by comparing fragmentation spectra from (derivatized) products with fragmentation spectra from authentic standards	N/A
1979^23^	5–21	≤0.08% yield of normal fatty acids (by mass, relative to initial CO)	GC-MS	Unclear how products were identified. Comparisons with authentic standards were not shown.	No data were shown to validate the FID procedure for quantifying product concentrations.
			GC-FID		
1997^41^	2	≤40% yield (by moles, relative to initial CH_3_SH)	GC-MS	Unclear how products were identified. Comparisons with authentic standards were not shown.	No data were shown to validate the GC-MS procedure for quantifying product concentrations.
1999^49^	6–22	≤20.8% of all products are fatty acids (by moles)	GC-MS	Unambiguous identification of select products by comparing fragmentation spectra and retention times with authentic standards.	No data were shown to validate the procedure for quantifying product concentrations.
			GC-FID		
2001^51^	7–18	≤20% yield of fatty acids (by mass, relative to total mass of reaction extract)	GC-MS	Unambiguous identification by comparing fragmentation spectra and retention times with authentic standards.	No data were shown to validate the GC-MS procedure for quantifying product concentrations.
2003^63^	2	None	Diffuse reflectance infrared Fourier transform spectroscopy	Using diffuse reflectance infrared Fourier transform spectroscopy, experimental spectra were compared to authentic standards.	N/A
2004^64^	10–13	C10: 53.7% yield	GC-MS	Unambiguous identification by comparing fragmentation spectra with authentic standards.	Calibration curves were described in the text to validate the procedure for quantifying product concentrations. Pentadecane was used as an internal standard for all compounds.
2004^52^	8–20	2.8% of all products are fatty acids (unclear whether by mass or moles)	GC-MS	Unambiguous identification by comparing fragmentation spectra and retention times with authentic standards.	No data were shown to validate the MS procedure for quantifying product concentrations.
2006^48^	2–4	C2: 1% yield	GC-MS	Unclear how products were identified. Comparisons with authentic standards were not shown.	No data were shown to validate the FID procedure for quantifying product concentrations.
		C3: 0.06% yield	GC-FID		
		C4: <0.02% yield			
		(by moles, relative to initial formic acid)			
2010^65^	1–2	C2: 9.0 mM	GC-MS	Unambiguous identification by comparing fragmentation spectra with authentic standards.	Calibration curves were shown to validate the procedure for quantifying product concentrations. Internal standards were not used.
		C3: 3.5 mM			
2016^66^	16, 18	C16: 0.011% yield	GC-MS	Unambiguous identification by comparing fragmentation spectra with authentic standards and literature references.	No data were shown to validate the MS procedure for quantifying product concentrations. Betulinic acid was mentioned as the internal standard for all products.
		C18: 0.02% yield			
		(by moles, relative to initial NH_2_CHO)			
2016^42^	Saturated: 3, 5	C3: 7.1 mM	GC-MS	For most products, unambiguous identification by comparing fragmentation spectra with authentic standards. For select products, identification was inferred on the basis of mass spectra and relative retention times.	Calibration curves were mentioned (but not shown) to validate the procedure for quantifying product concentrations. For some products, calibration curves were made with authentic standards. For other products, related compounds were used for calibration. Internal standards were not used.
		C5: 0.3 mM			
		C3 unsaturated: 3.9 mM			
	Unsaturated: 3, 5, 7, 9				
	C5 unsaturated: 7.9 mM				
	C7 unsaturated: 0.44 mM				
	C9 unsaturated: 0.011 mM				
2018^53^	7–26	7.8% of all products are fatty acids (unclear whether by mass or moles)	GC-MS	Unambiguous identification by comparing fragmentation spectra with authentic standards.	No data were shown to validate the GC-MS procedure for quantifying product concentrations. *N*-Eicosane-*d*_42_ was mentioned as the internal standard for all experiments.
2018^67^	1–2	Formic acid: ≤0.21% yield	GC-MS	Unambiguous identification by comparing GC-MS fragmentation spectra and retention times with authentic standards. In addition, H NMR spectra were compared with authentic standards.	H NMR calibration curves were shown to validate the procedure for quantifying product concentrations. Authentic standards were used, and DSS-Na was used as an internal standard.
		Acetic acid: ≤0.053% yield	^1^H NMR		
		(by moles, relative to initial CO_2_)			
2019^54^	2–10	36% of all 8-carbon products are fatty acids (by moles)	^1^H NMR	Unambiguous identification by comparing H NMR spectra with authentic standards.	Data from H NMR spectra were used to determine the relative abundance of fatty acids. Internal standards were not discussed.
2020^43^	1	C1: 1.5 μM	^1^H NMR	Unambiguous identification by comparing H NMR spectra with authentic standards.	H NMR spectra with an internal standard (acetone) were used to quantitate formate concentration. To validate the quantitation, additional formate was spiked in and a corresponding increase in H NMR signal was observed.
			13C-NMR		
2020^44^	1–2	C1: ∼20% yield	LC-MS	Unambiguous identification by comparing H NMR spectra with authentic standards.	H NMR calibration curves were mentioned (not shown) to validate the procedure for quantifying product concentrations. Authentic standards were used, and DSS-Na was used as an internal standard.
		C2: ∼0.4% yield			
		(by moles, relative to CO_2_)	LC-UV		
			^1^H NMR		
					
**Method 2: Electrochemistry**					
1967^72^	2–5	C2: 1.2% yield	GC-MS	Unambiguous identification by comparing fragmentation spectra and retention times with authentic standards.	No data were shown to validate the MS procedure for quantifying product concentrations.
		C3: 0.86% yield			
		C4: 0.088% yield			
		C5: 0.066% yield			
		(by moles, relative to initial CH_4_)			
1981^24^	2–7	C2: 0.10% yield	GC-MS	Unambiguous identification by comparing fragmentation spectra and retention times with authentic standards.	Calibration curves were shown to validate the procedure for quantifying product concentrations. Singly deuterated internal standards were used for each product.
		C3: 0.68% yield			
		C4: 0.014% yield			
		C5: 0.0050% yield			
		C6: 0.00073% yield			
		C7:0.00025% yield			
		(by moles, relative to initial CH_4_)			
2015^78^	1–2	C1: ∼1 μmoles	^1^H NMR	Unambiguous identification by comparing H NMR spectra with authentic standards.	H NMR calibration curves were shown (for formic acid) to validate the procedure for quantifying product concentrations. Authentic standards were used and Me_4_Si was used as an internal standard.
		C2: ∼0.5 μmoles			
2021^71^	6, 9	C6: ≤0.0078% yield	GC-MS	Unambiguous identification by comparing fragmentation spectra and retention times with authentic standards.	No data were shown to validate the GC-MS procedure for quantifying product concentrations.
		C9: ≤0.016% yield			
		(by mass, relative to total mass of reaction extract)			
					
**Method 3: Photochemistry**					
1960^79^	1–3	C1: 82 μmoles	Silica gel chromatography	Identification of products by comparison of retention times with authentic standards.	Silica gel chromatography was used to separate C1, C2, and C3 products. Each putatively pure product was titered with NaOH to find the equivalence point.
		C2: 234 μmoles			
		Acid–base titration			
		C3: 15 μmoles			
2019^54^	2–10	36% of all 8-carbon products are fatty acids (by moles)	^1^H NMR	Unambiguous identification by comparing H NMR spectra with authentic standards.	Data from H NMR spectra were shown and used to determine the relative abundance of fatty acids. Internal standards were not mentioned.
					
**Method 4: Irradiation by Massive Particles**					
1995^80^	13, 15, 17	C13: maximum 18.6 picograms	GC-MS	Unclear how products were identified. Comparison with authentic standards were not mentioned.	No data were shown to validate the MS procedure for quantifying product concentrations.
		C15: maximum 83.9 picograms			
		C17: maximum 57.0 picograms			
2010^81^	Undetermined	10^16^ molecules/cm^2^	FTIR	Regions of the FTIR spectra were considered diagnostic for carboxylic acids:	The intensity of the FTIR spectra at 1720 cm^–1^ was used to provide an upper limit on carboxylic acid concentration.
				ν(O–H) stretching = 3500–2500 cm^–1^.	
				ν(C=O) = 1720 cm^–1^	
				ν(C–O) = 1282 cm^–1^	
					
**Method 5: Gas-Phase Ion–Molecule Reactions**					
2003^82^	2–3	No data were shown	GC-MS	Unambiguous identification by comparing fragmentation spectra with authentic standards.	N/A
					
**Method 6: Redox Reactions in Solution**					
1963^83^	1	No quantitative data were shown	Paper chromat-ography	The presence of formic acid was inferred on the basis of the comparison of paper chromatography mobility to an authentic standard, and on the basis of a reaction with ammoniacal silver nitrate.	N/A
2000^85^	7 or 8	C7: 73–85% yield	GC-MS	Unambiguous identification by comparing fragmentation spectra with authentic standards.	No data were shown to validate the GC-MS procedure for quantifying product concentrations.
		C8: 65% yield			
		(unclear whether by mass or moles)			
2008^84^	1–3	C1: 7–20% yield	GC-MS	Unambiguous identification by comparing fragmentation spectra with publicly available data and by comparing retention times with authentic standards.	Calibration curves were mentioned (but not shown) to validate the procedure for quantifying product concentrations. An internal standard (heptadecane) was used.
		C2: 0.7–12% yield			
		C3: <0.01–0.2% yield			
		(by moles)			

a Unless otherwise specified, fatty acids are saturated and unbranched.

## Estimating the Contribution of Each Source to the Early Earth Fatty Acid Inventory

To gauge the relative importance of each fatty acid source for the origin of cells, we go beyond purely reviewing the literature with a goal of estimating how much fatty acid could be supplied to the early Earth from three sources: delivery by carbonaceous chondrites, catalysis by metal surfaces, and electrochemistry. Many relevant parameters for these estimates lack experimental constraints, so assumptions are needed. Our first assumption is that cells formed during the latter part of the Hadean eon; the full eon was ∼4.6 to 4.0 billion years ago (Gya).^18^ During the early Hadean, Earth was potentially hit by impactors that were large enough to sterilize the planet’s surface and reset the fatty acid inventory, so life must have originated after the last such event.^98^ The median age of estimates for the last ocean-vaporizing impact is ∼4.3 Gya,^99^ and we assume that life had originated by 4.0 Gya.^18^ In the subsections below, we construct back-of-the-envelope estimates for the total mass of fatty acids supplied to Earth during this interval. Our estimates are based on empirical data from the literature, and we indicate when existing models from the literature are applied. We articulate our assumptions and suggest experiments that will help to refine these estimates. Our estimates cannot distinguish fatty acids that form membranes (more than 8 carbons) from those that do not (less than 8 carbons) because the empirical data that one of our estimates relies on does not do so.

### Meteorite Delivery

We estimate the total mass of 2–12 carbon fatty acids delivered to Earth by carbonaceous meteorites, *M*_s_, using the following equation:
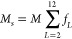
2In eq 2, *f*_*L*_ is the dimensionless fraction of a meteorite’s mass that comprises fatty acids of length *L*, where the length denotes the number of carbons in the fatty acid chain. Values for *f*_*L*_ depend on the type of carbonaceous meteorite, and average values can range from 10^–9^ for 10-carbon fatty acids to 10^–3^ for 2-carbon fatty acids.^26^ Parameter *M* is the time-integrated total mass of carbonaceous meteorites with radii of 1–100 m that would impact Earth from 4.3 Gya to 4.0. To estimate *M*, we integrate the following equation for the mass flux (kg/year) at time *t* (years) in the past from 4.3 to 4.0 Gya:
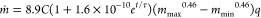
3This equation is adapted from Chyba and Sagan.^100^ In eq 3, *ṁ* is the mass flux (mass/year) of the total mass of carbonaceous meteorites with mass from *m*_min_ to *m*_max_ that would impact Earth per year; *C* is the frequency of carbonaceous meteorites relative to all types of meteorites (∼4%) observed in the meteorite fall record;^101^ τ is the decay constant of 144 million years for the impactor population; *m*_min_ is taken as the mass of a meteorite with a 1 m radius; *m*_max_ is taken as the mass of a meteorite with a 100 m radius; *q* is 1 kg^0.54^/year. We assume that all meteorites are spherical with a uniform density (2.1 g/cm^3^ for CM-type meteorites^38^ or 3.2 g/cm^3^ for C2-type meteorites^102^), so we can calculate the mass of a meteorite in kilograms from its radius in meters. Meteorites with radii less than 100 m can fragment into pieces that impact the Earth’s surface with low enough energy that fatty acids are preserved.^28,29^ Using eq 3, we estimate that ∼10^15^ kg of carbonaceous meteorites with radii between 1 and 100 m would impact Earth from 4.3 to 4.0 Gya.

Assuming that all carbonaceous meteorites are either CM1-type or C2-type, we use eqs 2 and 3 to calculate bounds for the total mass of fatty acids with a length of 2–12 carbons that could have been delivered to Earth from 4.3 to 4.0 Gya. We find that 10^10^–10^13^ kg of fatty acids with a length of 2–12 carbons could have been delivered to Earth from 4.3 to 4.0 Gya by carbonaceous meteorites. Our estimate varies over 3 orders of magnitude because the abundance of fatty acids on each type of meteorite (*f*_*L*_) varies considerably between different meteorite types. We consider only carbonaceous chondrites because they are the most well-characterized type of meteorite that can deliver fatty acids. We do not quantify additional uncertainties in the meteorite flux, nor do we quantify the influence of meteorite size, impact velocity, and impact angle on the fraction of fatty acids that survive impact.^103^

### Catalysis by Metal Surfaces after Vesta-Sized Impact

Next, we consider fatty acid synthesis on metal surfaces in the wake of a large Vesta-sized (∼10^20^ kg) asteroid impact on the early Earth. Such an impact would deliver iron to Earth’s surface, which could both act as a catalyst for fatty acid synthesis and generate a H_2_-rich atmosphere by reactions with steam from the vaporized ocean,^57^ as in eq 4:
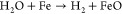
4An H_2_-rich atmosphere appears to be required for fatty acid synthesis by metal catalysts (Table 1). Such a massive asteroid impact would generate a high enough surface temperature to destroy most organic molecules on Earth; then, as the Earth cooled, the synthesis of fatty acids could occur in the H_2_-rich atmosphere. We consider only the last impactor that would reset Earth’s fatty acid inventory, which most likely hit Earth between 4.4 and 4.1 Gya.^99^ Significantly smaller meteorite impacts would not generate high partial pressures of H_2_ in the atmosphere because H_2_ escapes to space on time scales of ∼10^6^–10^7^ years. Additionally, small impactors would not generate global surface temperatures above 200 °C, which we assume is the minimum temperature required for fatty acid synthesis. Therefore, smaller impactors would not produce many fatty acids, and we do not consider their contribution here.

To estimate the total mass of fatty acids synthesized by metal catalysts after such an impact, *M*_c_, we assume that the rate of fatty acid synthesis depends linearly on gas pressures according to the following equation:
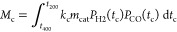
5Here, *k*_c_ is an empirical rate constant for fatty acid synthesis, calculated from the results of experiments at 400 °C by Nooner and Oro.^23^ They provide the only measurement to date of the absolute concentration of fatty acids produced on a metal catalyst in the absence of an aqueous phase. The value of *k*_c_ is 7.4 × 10^–6^ kilograms of 6–18 carbon fatty acids (excluding 12-carbon fatty acids, for which data are not available) per bar of H_2_, per bar of CO, per hour of reaction time, per kilogram of catalytic surface available.^23^ Only the summed mass of all fatty acids is reported by Nooner and Oro;^23^ there is no information given about the number of moles of individual fatty acids. Here, *m*_cat_ is the mass of available metal catalyst, in kilograms. *P*_H2_ and *P*_CO_ are the atmospheric partial pressures of hydrogen and carbon monoxide, respectively, in bar; both are functions of time, *t*_c_. The product is integrated over time from *t*_400_ to *t*_200_. *t*_400_ is the number of hours after the impact until the surface temperature reaches 400 °C, and *t*_200_ is the number of hours after the impact until the surface temperature cools to 200 °C. We assume that fatty acid synthesis occurs at a constant rate *k*_c_ when the temperature is within this range and that the reaction does not occur when the temperature is outside this range.

We assume that 33% of the asteroid’s mass is iron and that 7% of this iron (i.e., 2.3% of the total asteroid mass) remains available on Earth’s surface to catalyze fatty acid synthesis (giving *m*_cat_ = 2.3 × 10^18^ kg) based on a linear extrapolation of the impact simulation performed by Citron and Stewart.^56^ The assumption of ∼33% iron mass follows Zahnle et al.,^55^ which is the total iron in high iron enstatite (EH-type) meteorites^104^ and also the fraction of Earth’s mass in its iron core.^105^ Bodies with enstatite composition are candidates for impactors that hit the Earth after the Moon-forming impact and Earth’s core formation, although the contribution of carbonaceous versus enstatite compositions is debated.^106−108^ In EH enstatites, which are highly reduced, most of the iron is metallic with a smaller fraction of iron sulfide.^109^ In a postimpact vapor plume, the iron is vaporized into atoms, which condensation sequences show condenses to form metallic iron with subsequent cooling.^110^

We assume that another fraction of the asteroid’s iron (between 1% and 90%) is used to generate H_2_ from excess H_2_O according to eq 4. We adapted a thermochemical model previously developed by Zahnle et al. to compute *P*_H2_, *P*_CO_, and temperature as a function of time after the impact.^55^ Depending on the fraction of the asteroid’s iron that reacts with water to produce H_2_ gas, we find that *P*_H2_ ranges from 10^–3^ to 10^–1^ bar and *P*_CO_ ranges from 10^–3^ to 10^–7^ bar. We calculate that the Earth’s postimpact temperature would be between 200 and 400 °C for ∼1000 years, and we assume that fatty acid synthesis occurs at a constant rate during this time. Equation 5 estimates that between 10^11^ and 10^15^ kg of fatty acids with a length 6–18 of carbons (excluding 12 carbons, for which data are not available) would be synthesized using metal catalysts in the wake of a Vesta-size impactor.

Our estimate for the total mass of fatty acids synthesized by metal catalysts is uncertain because many factors in our analysis are poorly constrained. First, it is not known whether reduced metals would remain exposed to the atmosphere on Earth’s surface after an extremely massive impact.^56^ Additionally, it is unclear how a H_2_O steam atmosphere would influence the rate of fatty acid synthesis; steam can affect the yield of Fischer–Tropsch reactions in different ways, depending on the type of catalyst and the type of reaction product.^111^ Even without steam in the atmosphere, it is uncertain whether the rate of fatty acid synthesis depends linearly on gas partial pressures. Kinetic models for the Fischer–Tropsch process have been developed in industrial settings, which generally do not mimic plausible early Earth conditions; functions for each partial pressure depend on the engineered design of the catalyst.^58^ Additionally, the effect of temperature on the final product distribution also appears to depend on the catalyst design.^51,112^ Separate experiments indicate that the yield of fatty acids depends linearly on the amount of catalyst surface area that is available.^64^ In general, further experiments are necessary to constrain these reaction parameters and the precise functional form for fatty acid production in plausible conditions on the early Earth.

### Electrochemical Synthesis

We also construct an estimate for the total mass of fatty acids that could be synthesized by electrochemistry from 4.3 to 4.0 Gya,
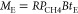
6where *R* is an empirical rate constant for the synthesis of 2–7 carbon fatty acids, calculated from electrochemical experiments by Yuen et al.^24^ The value of *R* is 1.1 × 10^–17^ kilograms of 2–7 carbon fatty acids per bar of CH_4_, per joule of electrical energy dissipated by sparking, per hour of reaction time.^24^ Although there are no electrochemical experiments that provide absolute concentrations for membrane-forming (longer than 8 carbons) fatty acids, Yuen et al.^24^ provide absolute concentrations for the largest number of fatty acids. *P*_CH4_ is the partial pressure of methane on the early Earth, which we estimate to be between 10^–15^ to 10^–1^ bar after the last Vesta-sized (∼10^20^ kg) asteroid impact. This value depends on the initial preimpact abundance of atmospheric CO_2_, the fraction of the impactor’s iron that becomes oxidized (1–100%), and the importance of methane-forming catalysts, which may reduce the quench temperature of methane, thereby increasing its abundance.^55^*B* is the amount of electrical energy dissipated by lightning and corona discharges on the Hadean Earth during a year. We assume that *B* is 1.5 × 10^18^ joules per year, which is the electrical energy dissipated per year on the modern Earth.^76^*t*_E_ is the reaction time in years. We assume *t*_E_ is 10^5^ years, which is an estimate for the lifetime of a methane-rich atmosphere after a Vesta-sized (∼10^20^ kg) asteroid impact.^55^

By applying these assumptions, we calculate that 10^–4^ to 10^10^ kg of fatty acids with a length of 2–7 carbons could be synthesized on Earth from 4.3 to 4.0 Gya by electrochemical reactions. Although separate experiments have shown that electrochemical production of membrane forming fatty acids with more than 8 carbons is possible,^71^ membrane-forming fatty acids were not detected in the experiments by Yuen et al.,^24^ so our estimate is not directly informative about membrane formation on early Earth. More data are needed about the absolute concentration of membrane-forming fatty acids produced during electrochemical experiments.

There are uncertainties in our electrochemistry estimate. First, there is insufficient information available to estimate the total energy dissipated by the Tesla coil during the experiments by Yuen et al.^24^ If we assume that their Tesla coil used 30 000 V potential^71^ and 15 A, we can estimate that ∼4 × 10^10^ joules of energy were dissipated during their 24 h experiment. Even if we had perfect information about the Tesla coil voltage and current, it might not be straightforward to estimate the amount of energy that is usable for chemical synthesis.^113^ Furthermore, we assumed that the yield of fatty acids depends only linearly on the amount of available electrical energy.^76^ Further experiments are necessary to validate this assumption. Experiments by Schlesinger and Miller suggest that the yield of amino acids during sparking does increase linearly with increasing CH_4_ partial pressure (below ∼ 0.06 bar),^77^ but further experiments are needed to validate this assumption for fatty acids.

### Summary of Estimates

We have compared the total mass of fatty acids produced by 3 different sources during the Hadean eon, following the last extremely massive impactor that would have reset Earth’s fatty acid inventory. We estimate that 10^11^ to 10^15^ kg of 6–18 carbon fatty acids could have been synthesized by metal catalysts derived from the massive impactor. The total mass of fatty acids that could have been delivered by carbonaceous meteorites is 10^10^ to 10^13^ kg of 2–12 carbon fatty acids. The yield of 2–7 carbon fatty acids from electrochemical processes is potentially smaller, between 10^–4^ and 10^10^ kg. Consequently, an integrated supply of fatty acids to the Earth’s surface from all sources (dominated by metal surface production) between ∼10^–4^ and ∼10^0^ kg/m^2^ is possible, given the Earth’s surface area of 5.1 × 10^14^ m^2^.

Ultimately, the local concentration of fatty acids determines whether or not membranes form, so the possible sources should be evaluated by this criterion. Although meteorites could have delivered a significant mass of fatty acids across the Earth’s surface, the aqueous concentration of fatty acids in a single waterbody would not have been high enough to form membranes without evaporating a significant volume of water (Figure 3). In contrast, a local stockpile of fatty acids could have been produced on atmosphere-exposed metal surfaces after an extremely massive impact, and subsequent dissolution into water could have allowed membrane formation. Although little is known about the electrochemical synthesis rate for membrane-forming fatty acids, repeated lightning strikes into the same small waterbody seem unlikely, so it is unclear whether a high enough local concentration of membrane-forming fatty acids could have formed via electrochemistry. Fatty acids in aqueous solution can be degraded via photochemistry,^114^ so fatty acids that are slowly synthesized by electrochemistry may not have attained high enough concentrations to form membranes, whereas a large stockpile of fatty acids dissolving off metal surfaces may have been less sensitive to photochemical degradation.

Although the estimates above have many uncertainties, they are valuable as a first attempt to quantitatively compare fatty acid sources on the early Earth. We hope that future experiments can further constrain these estimates.

## Alternative Amphiphiles

In addition to fatty acids, alternative types of amphiphiles may have been synthesized on the early Earth,^115−117^ and these amphiphiles might have incorporated into the membranes of the earliest cells. For example, alcohols are commonly produced along with fatty acids in many experiments,^48,49,51−54^ and long-chain fatty alcohols are known to stabilize fatty acid membranes.^5,118^ An excess of long-chain fatty alcohols form oil droplets, and the oil may disrupt membranes. In addition, phase-separated coacervates could have served as another type of prebiotic compartment,^119,120^ and fatty acid membranes may have even assembled around such coacervate compartments.^121^

## Conclusions

Fatty acids can assemble into membranes and could have formed the boundaries for the first cells. Our Review highlights multiple potential sources of fatty acids on the early Earth. The three most well-characterized sources are meteorite delivery, synthesis on metal surfaces, and synthesis by electrochemistry. Other reactions involving photochemistry, irradiation by massive particles, ion–molecule reactions, and diverse redox reactions in aqueous solution may have also produced fatty acids in natural environments. To refine quantitative estimates for the relative importance of each fatty acid source, more detailed constraints are needed. We highlight the following questions to help any future experiments have the widest possible impact:

- How do the yields of fatty acids from metal-catalyzed reactions depend on the temperature and the partial pressures of gases such as H_2_, CO_2_, and H_2_O? Data would help to constrain estimates for fatty acid production following ocean-vaporizing impacts.
- Can membrane-forming fatty acids (>8 carbons) form in hydrothermal experiments with nonoxidized, ultramafic minerals as the sole catalyst? Data would elucidate the potential for fatty acid production at hydrothermal vents.
- How do the yields of fatty acids depend on the voltage, duration, or total energy dissipated during electrical sparking? Data would help to constrain estimates for fatty acid production during lightning strikes.
- What are the absolute concentrations of fatty acids in these types of experiments?

In summary, our analysis suggests that fatty acids could have been available on the early Earth. We have not assessed whether those fatty acids would have been sufficiently concentrated to assemble into membranes except in the limited case of small meteorite fragments delivered into aqueous environments. For fatty acids supplied via alternative sources, further data are required to assess the potential for membrane formation. By investigating possible sources of fatty acids on the early Earth, we hope to constrain the environmental setting for the origin of cells.
